# Intermittent pain of the pelvis in a Syrian woman

**DOI:** 10.1371/journal.pntd.0008183

**Published:** 2020-06-18

**Authors:** Benjamin T. Schleenvoigt, Bernhard Theis, Michaela Wüst, Christina Forstner, Mathias W. Pletz, Stefan Hagel

**Affiliations:** 1 Institute for Infectious Diseases and Infection Control, Jena University Hospital, Jena, Germany; 2 Section Pathology of the Institute of Forensic Medicine, Jena University Hospital, Jena, Germany; 3 Department of Internal Medicine IV (Gastroenterology, Hepatology, and Infectious Diseases), Jena University Hospital, Jena, Germany; 4 Department of Medicine I, Division of Infectious Diseases and Tropical Medicine, Medical University of Vienna, Vienna, Austria; Ben-Gurion University of the Negev, ISRAEL

## Presentation of case

**Fig 1:**
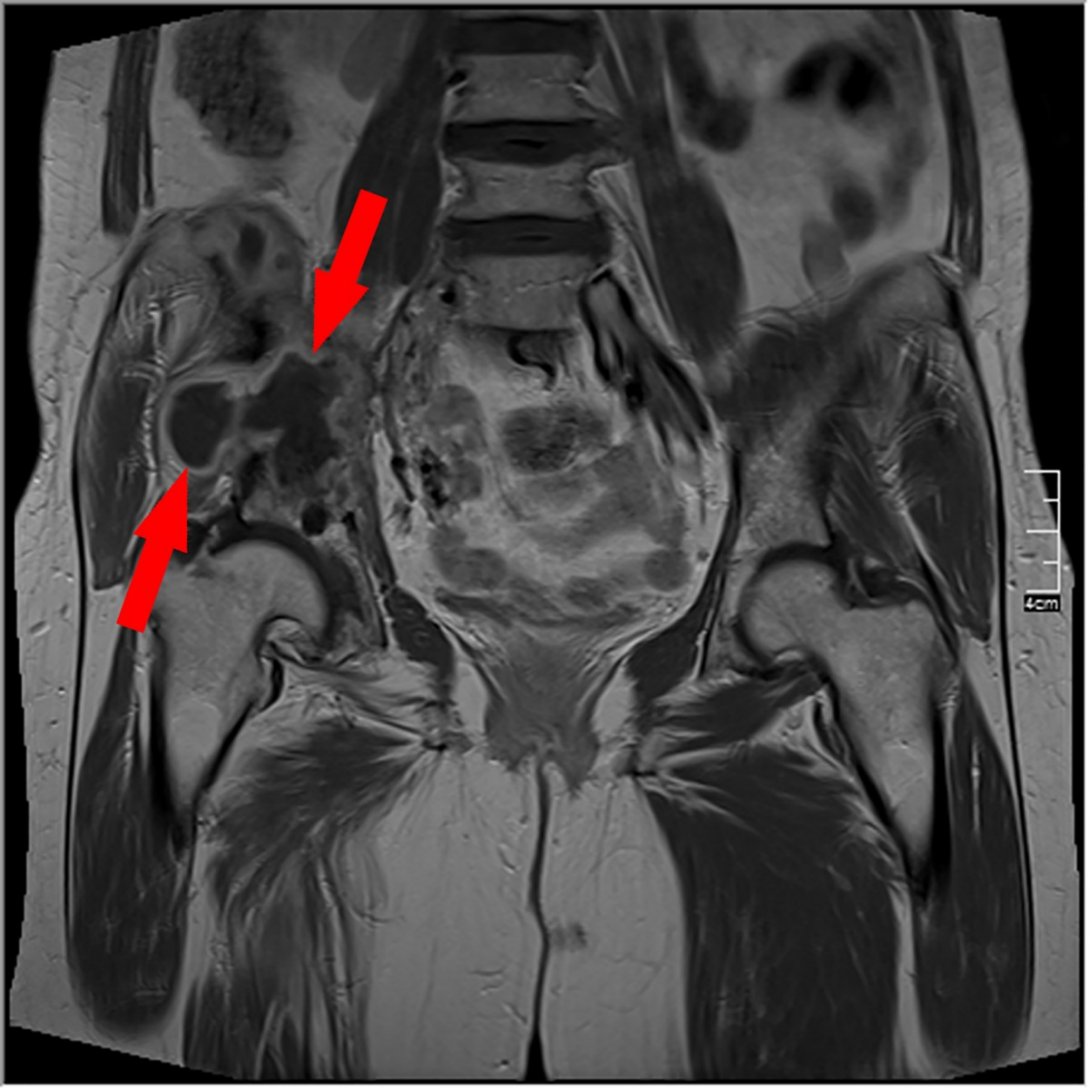
Cystic formation with intra- and extrapelvic parts as well as migration of the right Ala ossis ilii and sacroiliac joint (T1 post-contrast agent). *Image credit*: *S*. *Gießler*. T1, T1-weighted imaging.

**Fig 2:**
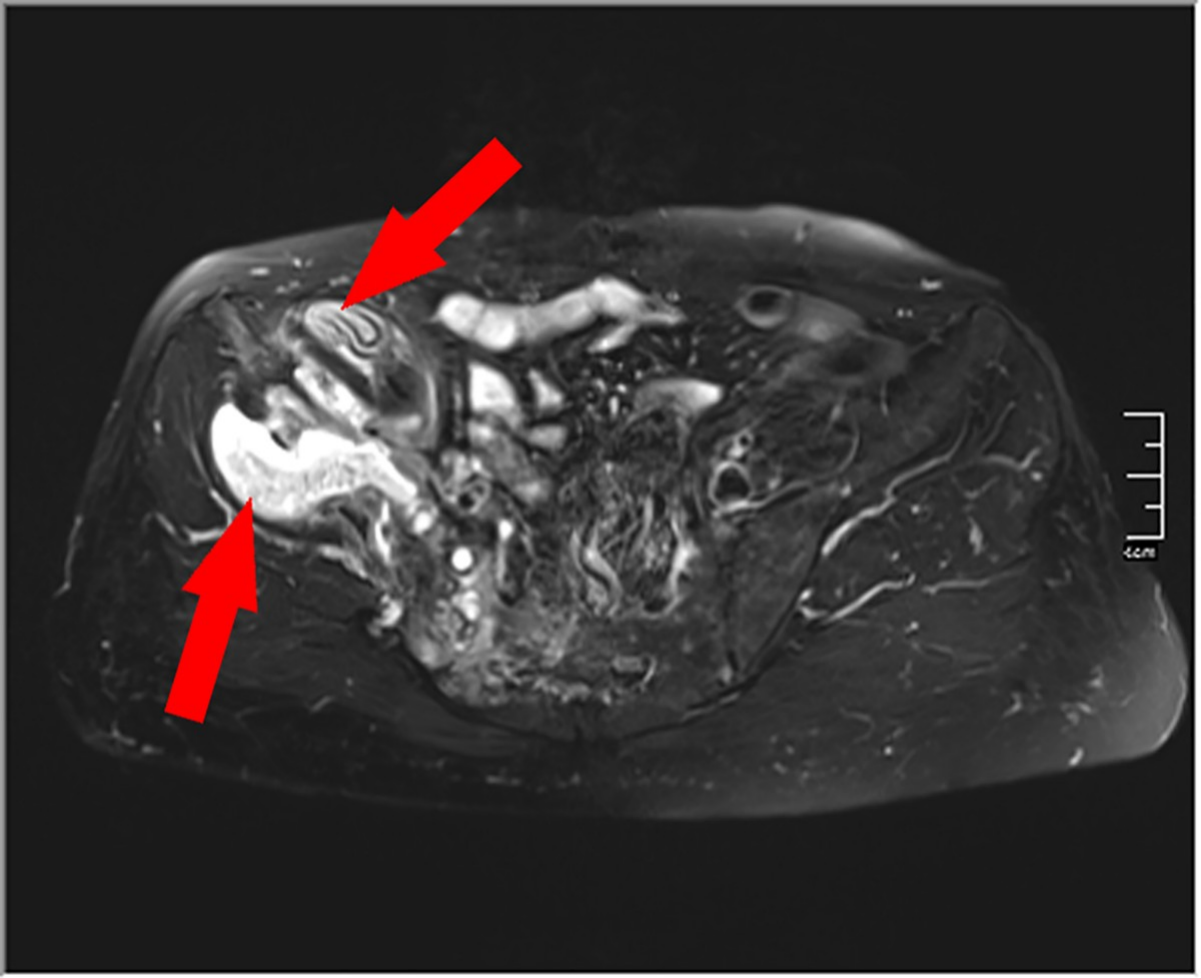
Cystic formation with intra- and extrapelvic parts as well as migration of the right Ala ossis ilii and sacroiliac joint (T2). *Image credit*: *S*. *Gießler*. T2, T2-weighted imaging.

**Fig 3:**
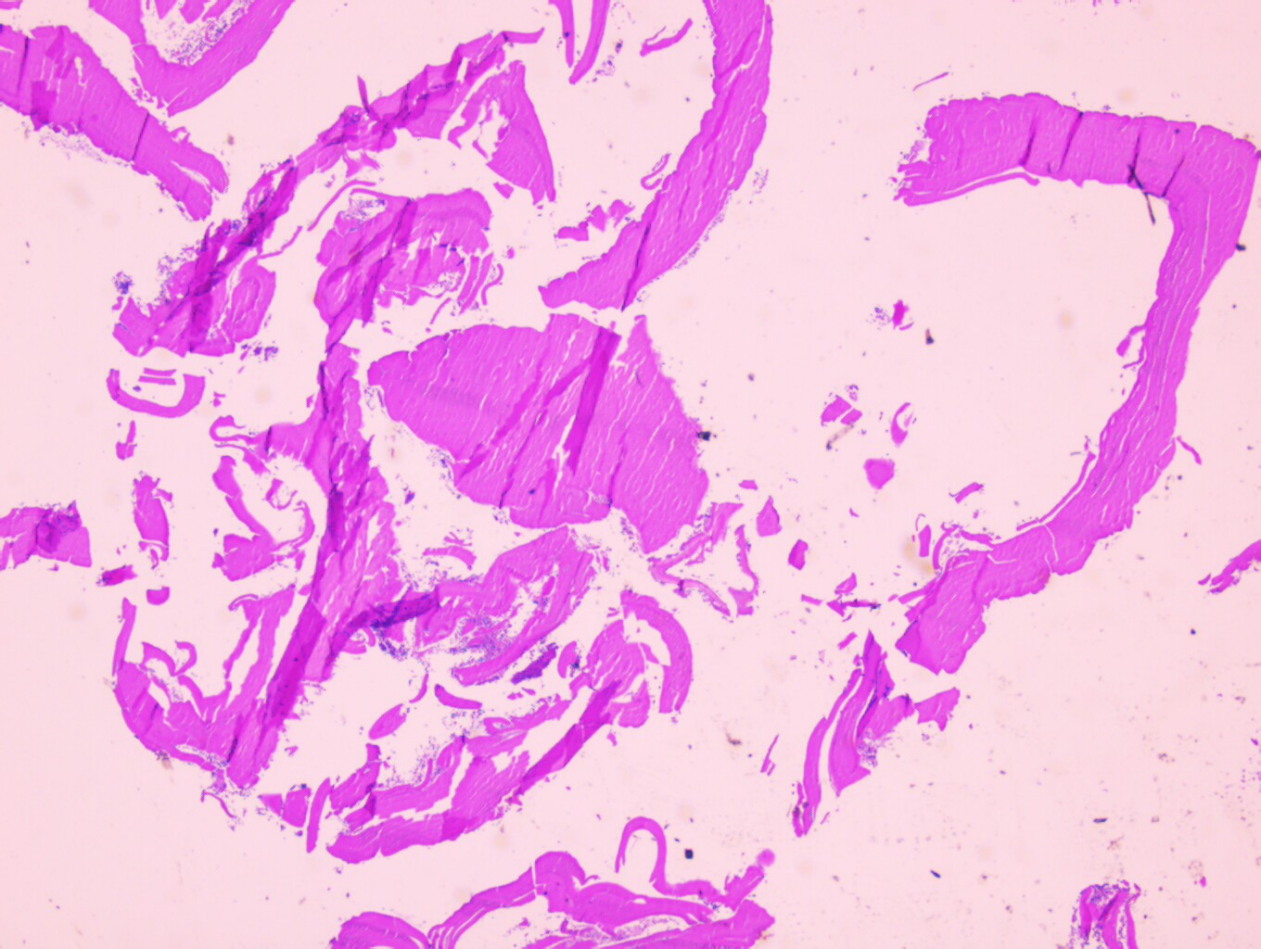
Numerous fragments of the hydatid cyst wall at low magnification, 25x (HE stain). *Image credit*: *B*. *Theis*. HE, hematoxylin–eosin.

**Fig 4:**
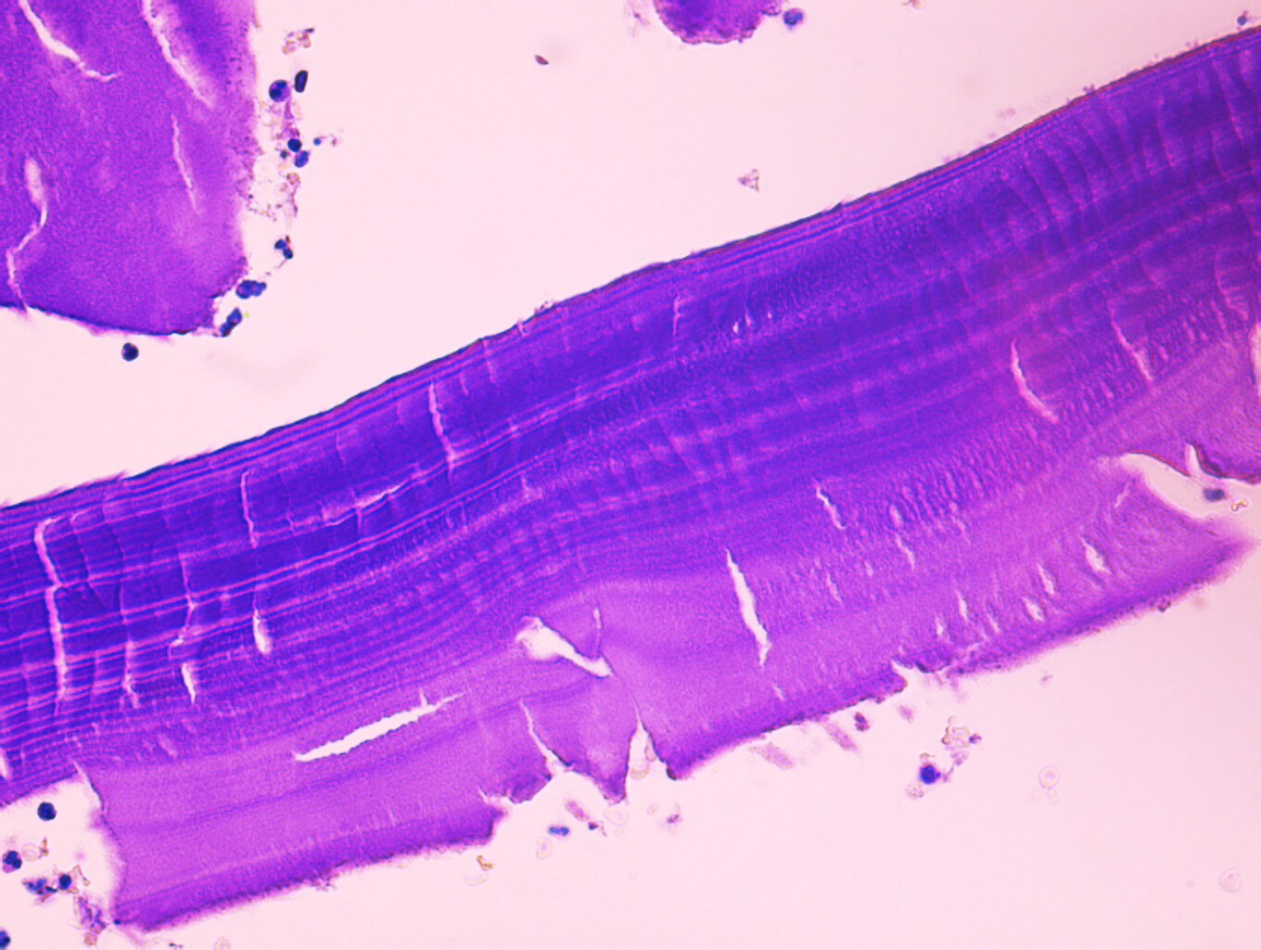
Characteristic lamellar membranous structure of the hydatid cyst wall, 400x (PAS stain). Neither a well-defined germinal epithelium, nor protoscolices were observed, which may indicate an infertile state of the hydatid cyst. *Image credit*: *B*. *Theis*. PAS, Periodic acid–Schiff.

A 66-year-old immunocompetent woman from Syria presented with a two-months history of intermittent pain of the right pelvis to the Jena University Hospital (Germany). Fever, night sweats and weight loss were denied. An MRI scan of the pelvis showed marked destruction of os ilium and os sacrum and a sinus tract draining in multiple subcutaneous cystic lesions (Fig 1 and Fig 2). Erythrocyte sedimentation rate was slightly elevated (30 mm/h), and C-reactive protein and white blood cell count were normal. There was no eosinophilia or Immunoglobulin E (IgE) elevation. To establish diagnosis, a bone biopsy was performed. Histopathology showed loose edematous connective tissue, a mixed inflammatory infiltrate, and regressive calcifications. PAS-positive cuticular components with typical lamellar layering, consistent with echinococcosis were detectable. Furthermore, wall fragments of cystic lesions were visible (Fig 3 and Fig 4). Immunohistochemistry directed against specific antigens of *Echinococcus granulosus* and *E*. *multilocularis* confirmed the former as causative agent. Due to the localization and extensive bone destruction, no surgical excision was possible. Consequently, empirical treatment for tuberculosis was stopped, and therapy with albendazole (400 mg twice daily) was initiated for an indefinite period.

## Case discussion

Echinococcosis is a neglected tropical disease recognized by the World Health Organization (WHO). Cystic Echinococcosis (CE) is caused by metacestodes—the larval stage of the species *E*. *granulosus*. Adult parasites live in the intestine of the definitive host. These are dogs and other carnivores. The intermediate hosts—usually sheep and other herbivores—get infected by oral intake of the eggs. The human is an intermediate incidental host who can harbor the vesicular metacestode in any anatomical site. The liver is the most frequent location of the fluid filled cystic lesions (70%), followed by the lungs (20%). The central nervous system is affected in 3%. Osseous echinococcosis is a rare disease (1% to 4%). It can involve any bone, but has a predilection for the vertebral column (45%), pelvis (14%), and femur (10%) [1,2]. Multiosseous infestation and concomitant soft tissue or organ involvement are frequent, particularly the combination of proximal femur involvement with the pelvic girdle. In a small study from Kazakhstan including 8 patients with CE of the bone, the pelvis was the predominant localization. In all cases, malignancy was initially suspected [3].

The prevalence of CE in the population of migrants from Middle East countries is not known. Diagnoses relies on imaging, in addition to serology, and occasionally microscopic identification of tissue samples. Ultrasound is the cornerstone for diagnosis, staging, and follow-up. It can be supplemented by computed tomography (CT) and MRI. When ultrasound is unavailable, MRI should be the preferred cut imaging [4]. Diagnosis is often protracted as CE of the bone lacks a typical clinical appearance and image characteristics on X-ray or CT scan are similar to those of tuberculosis, metastases, and giant cell tumors or bone cysts. Complete surgical excision of the lesions combined with albendazole chemotherapy are the standard of care with a 17% risk of recurrence [5]. The dual therapeutic combination of albendazole with praziquantel seems to be more effective than albendazole alone and is occasionally used for therapy of CE [6]. In most cases, complete surgical intervention is not possible and thus the prognosis is very poor. Frequent sequels of the highly destructive parasitic process are chronic pain, pseudarthrosis, fractures, and death [5, 7, 8].

Physicians should be aware of this rare differential diagnosis in cystic bone lesions, especially in migrating populations.

**Key learning points**

- CE is an important differential diagnosis of cystic bone lesions, especially in migrating populations.
- CE can involve any bone, but has a predilection for the vertebral column (45%), pelvis (14%), and femur (10%).
- In histopathology, PAS-positive cuticular components with typical lamellar layering of the hydatid cyst can be observed in CE.
- If possible, surgical excision of the lesions combined with albendazole chemotherapy are standard of care for CE of the bone.

## References

[1] Agudelo Higuita NI, Brunetti E, McCloskey C. Cystic Echinococcosis. J Clin Microbiol. 2016;54(3):518–23. 10.1128/JCM.02420-15 26677245 PMC4767951

[2] Neumayr A, Tamarozzi F, Goblirsch S, Blum J, Brunetti E. Spinal cystic echinococcosis—a systematic analysis and review of the literature: part 2. Treatment, follow-up and outcome. PLoS Negl Trop Dis. 2013;7(9):e2458 10.1371/journal.pntd.0002458 24069501 PMC3777903

[3] Manciulli T, Mustapayeva A, Juszkiewicz K, Sokolenko E, Maulenov Z, Vola A, et al Cystic Echinococcosis of the Bone in Kazakhstan. Case Rep Infect Dis. 2018;2018:9682508 10.1155/2018/9682508 30319824 PMC6167588

[4] Stojkovic M, Rosenberger K, Kauczor HU, Junghanss T, Hosch W. Diagnosing and staging of cystic echinococcosis: how do CT and MRI perform in comparison to ultrasound? PLoS Negl Trop Dis. 2012;6(10):e1880 10.1371/journal.pntd.0001880 23145199 PMC3493391

[5] Steinmetz S, Racloz G, Stern R, Dominguez D, Al-Mayahi M, Schibler M, et al Treatment challenges associated with bone echinococcosis. J Antimicrob Chemother. 2014;69(3):821–6. 10.1093/jac/dkt429 . 24222611

[6] Velasco-Tirado V, Alonso-Sardon M, Lopez-Bernus A, Romero-Alegria A, Burguillo FJ, Muro A, et al Medical treatment of cystic echinococcosis: systematic review and meta-analysis. BMC Infect Dis. 2018;18(1):306 10.1186/s12879-018-3201-y 29976137 PMC6034244

[7] Cattaneo L, Manciulli T, Cretu CM, Giordani MT, Angheben A, Bartoloni A, et al Cystic Echinococcosis of the Bone: A European Multicenter Study. Am J Trop Med Hyg. 2019;100(3):617–21. 10.4269/ajtmh.18-0758 30693857 PMC6402919

[8] Monge-Maillo B, Chamorro Tojeiro S, Lopez-Velez R. Management of osseous cystic echinococcosis. Expert Rev Anti Infect Ther. 2017;15(12):1075–82. 10.1080/14787210.2017.1401466 . 29110551

