# Correction: Intermittent pain of the pelvis in a Syrian woman

**DOI:** 10.1371/journal.pntd.0008569

**Published:** 2020-08-03

**Authors:** Benjamin T. Schleenvoigt, Bernhard Theis, Michaela Wüst, Christina Forstner, Mathias W. Pletz, Stefan Hagel

## Notice of Republication

This article was republished on June 25, 2020, to remove a Supporting Information file that was incorrectly included in the originally published article. The main article text and figures are unchanged. Please download this article again to view the correct version.
